# Perinatal HIV transmission and the cost-effectiveness of screening at 14 weeks gestation, at the onset of labour and the rapid testing of infants

**DOI:** 10.1186/1471-2334-8-174

**Published:** 2008-12-31

**Authors:** Belinda Udeh, Chiedozie Udeh, Nicholas Graves

**Affiliations:** 1 Public Policy Center, University of Iowa, Iowa City, Iowa, USA; 2 University of Iowa Hospitals and Clinics, University of Iowa, Iowa City, Iowa, USA; 3 Institute of Health & Biomedical Innovation, Queensland University of Technology, Brisbane, Queensland, Australia

## Abstract

**Background:**

Preventing HIV transmission is a worldwide public health issue. Vertical transmission of HIV from a mother can be prevented with diagnosis and treatment, but screening incurs cost. The U.S. Virgin Islands follows the mainland policy on antenatal screening for HIV even though HIV prevalence is higher and rates of antenatal care are lower. This leads to many cases of vertically transmitted HIV. A better policy is required for the U.S. Virgin Islands.

**Methods:**

The objective of this research was to estimate the cost-effectiveness of relevant HIV screening strategies for the antenatal population in the U.S. Virgin Islands. An economic model was used to evaluate the incremental costs and incremental health benefits of nine different combinations of perinatal HIV screening strategies as compared to existing practice from a societal perspective. Three opportunities for screening were considered in isolation and in combination: by 14 weeks gestation, at the onset of labor, or of the infant after birth. The main outcome measure was the cost per life year gained (LYG).

**Results:**

Results indicate that all strategies would produce benefits and save costs. Universal screening by 14 weeks gestation and screening the infant after birth is the recommended strategy, with cost savings of $1,122,787 and health benefits of 310 LYG. Limitations include the limited research on the variations in screening acceptance of screening based on specimen sample, race and economic status. The benefits of screening after 14 weeks gestation but before the onset of labor were also not addressed.

**Conclusion:**

This study highlights the benefits of offering screening at different opportunities and repeat screening and raises the question of generalizing these results to other countries with similar characteristics.

## Background

Perinatal transmission causes most HIV infection among new born infants [1]. Transmission occurs during pregnancy, at the time of delivery or through breast milk [2]. The risk of perinatal HIV transmission can be reduced from 13–43% to less than 2% [3-5] if an accurate diagnosis is made and appropriate treatment provided. Universal antenatal screening for HIV in pregnancy is now advocated in a number of countries [6,7] and a number of cost-effectiveness studies have been published [8-12]. These describe high income settings where HIV prevalence is low, prenatal care is widely available and adherence is good and typically screening is offered once during the 1^st ^trimester [8,9,13]. There is also literature that describes low income countries where the HIV prevalence is high but health resources are quite scarce making treatment options limited after diagnosis [10,11]. There is little research for settings where prevalence is relatively high and adherence to prenatal care is low, yet resources are available to make an accurate diagnosis and deliver effective therapy. In this case there is a clinical rationale for offering a diagnostic test to women who present in the first trimester and again at the onset of labour and for the rapid testing of infants. The process of repeat screening will overcome the problem of poor adherence to prenatal care and also captures women who may not have seroconverted at the time of the first test and women who contract the virus after the first screen. There is evidence that risks of perinatal transmission can be mitigated if a diagnosis is made at the onset of labor [14,15] and if the infant is screened [2,16], however, repeat screening regimens will incur costs and these must be balanced against health benefits.

In the U.S. Virgin Islands, less than 70% of women received antenatal care within the first trimester and of these women, less than 30% are screened for HIV [17,18]. The aim for this study is to develop a model of the incremental costs and incremental health benefits of nine different combinations of perinatal HIV screening strategies compared to existing practice. Three screening opportunities and all logical combinations are evaluated. These include screening by 14 weeks gestation during prenatal care, screening at the onset of labour and screening of the infant within 24 hours of birth. An alternative to the Enzyme Linked Immunoassay (EIA) screening tests is also considered, this is a rapid point of care test (Orasure^®^). The setting is the U.S. Virgin Islands which is an upper income setting, although on the low end of the World Bank classification scale (GDP US$15,000 per capita [19]). It has a population of around 110,000, over 1500 births per year [20], and has relatively high prevalence of antenatal HIV. It is a U.S. territory with many of its health policies adopted from the mainland U.S. even though its population characteristics and resource availability is considerably different. The results from this study can be generalized to other settings where prevalence is relatively high, adherence to prenatal care is poor yet resources are available for screening and treatment.

## Methods

### Overview

**Figure 1:**
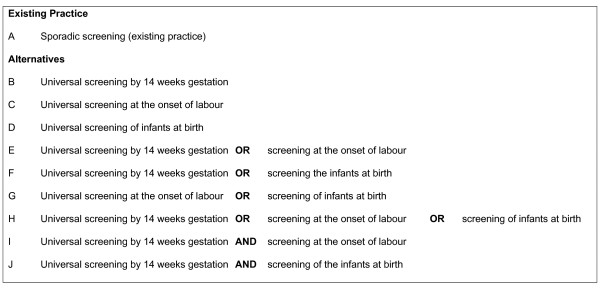
**Existing Practice and the Nine Alternate Models of Perinatal HIV Screening**.

A cost-effectiveness decision-analytic model was built using TreeAge Pro^®^. A societal perspective was adopted to include the relevant health service costs, private costs and production losses. The expected incremental costs and health benefits, expressed as life years gained, of all logical alternate screening strategies were compared to existing practice at the decision node. See Figure 1 for a list of the strategies evaluated. The following uncertain events were built into the tree: HIV negative versus HIV positive woman; woman seeks prenatal care versus doesn't seek prenatal care; woman accepts screening versus declines screening; HIV test positive versus negative; pregnancy terminated versus not terminated; woman accepts ART therapy versus declines ART; HIV averted in infant versus HIV positive infant. All cost and benefit data was collected for a reference year of 2004 and were counted in $US. Data values were verified by local experts where relevant. All future costs and benefits were discounted at a rate of 3% in line with current recommendations [21]. Ethical permission for the study was granted by the Queensland University of Technology Research Ethics Committee.

### Incremental Costs

Values for all relevant costs were included. The incremental cost of implementing the screening program included the costs of treating true HIV positive and false HIV positive mothers while pregnant, the treatment associated with early diagnosis in HIV positive mothers after delivery and HIV positive children, and the avoided treatment costs from preventing a case of HIV. The cost savings were deducted from all other positive costs to derive a net cost of the alternate screening strategy. It was assumed that all women would receive pre and post test counseling. An EIA test would be used for women who were screened by 14 weeks during antenatal care and rapid point of care test (Orasure^®^) for those women screened at the onset of labour or those infants screened within 24 hours birth. All positives were confirmed with a western blot. For false positives from rapid testing, treatment would be offered because a confirmation of HIV status would not be available before treatment would have to be commenced. For all infants born to HIV positive mothers, confirmation of their HIV status would be through the use of a HIV Polymerase Chain Reaction (PCR) test at time intervals in line with the U.S. Preventive Services Taskforce Guidelines [2]. All true positives and false positives identified at the onset of labor or by screening the infant after birth would begin appropriate medical therapy in accordance with current guidelines including prophylaxis for infants born to HIV positive mothers [7]. Medical treatment costs would also be included for all HIV positive women up until the time they would have been diagnosed by other means, with similar costs included for all infants who contract HIV despite screening and prophylaxis up until the time they would have otherwise been diagnosed. The lifetime costs of treating a case of HIV were determined from the rate of disease progression of the infant. For each case of HIV avoided, the lifetime costs of treatment and care giving that would have been incurred were deducted from the total costs of screening and treatment to derive the net cost of the strategy.

### Incremental Benefits

Incremental benefits were measured in life years gained (LYG). These included the LYG from avoided HIV infection in infants, and the LYG from earlier detection and treatment of HIV positive mothers and HIV positive infants. In the repeat screening strategies (i.e. E, F, G, H, I & J) the conservative assumption was made that if a woman refused a first test offer, she would refuse all subsequent test offers. Using the prevalence of HIV and the sensitivity and specificity of the screening test as determined from published literature, the number of true positives and false positives were estimated. It was assumed that not all women diagnosed with HIV would accept antiretroviral therapy (ART) for themselves but would comply with the recommendations for infant prophylaxis, not breastfeeding their infant, and treatment if their infant is HIV+. The ART and prophylaxis protocols for both mothers and infants corresponds to the recommendations from U.S. Preventive Services Taskforce [7]. Rates of perinatal HIV transmission determined from the literature were applied to the number of true positives identified to determine the number of cases of HIV avoided. According to current research, some HIV positive infants would be 'fast' progressors while others would be 'normal' progressors [22-24]. The net LYG would be the number of LYG from an avoided case of HIV in an infant adjusted for their expected rate of disease progression had they contracted HIV, the LYG from the earlier diagnosis of a HIV positive mother and, the earlier diagnosis of an infant who still contracts HIV despite screening and prophylaxis also adjusted by their expected rate of disease progression.

### Uncertainty and sensitivity analyses

For all variables, the base case, low and high values and the source are presented in Tables 1, 2, 3, 4. The variables for which the model results were most sensitive were identified through a series of one-way sensitivity analyses and this information was summarized in a tornado diagram. These variables were rigorously tested using a threshold and two way sensitivity analysis. To further test the robustness of results to uncertainty within the model, a microsimulation was performed (n = 10,000 microsimulations). The intention of a microsimulation is to demonstrate variability in a population and therefore test first order uncertainty in the model. It involves running one patient at a time through the model with the events based on the underlying probabilities in the model. The costs, effects and net monetary benefits are calculated for each run through the model for each strategy.

**Table 1:** Cost Variables (Labour, Counseling & Testing, Training and Health Promotion) used in model with 'baseline', 'low' and 'high' values, and sources

**Variable**	**Baseline (Low, High)**	**Source**
Monthly laboratory technician salary	$2974 ($2230, $3717)	[33]
Monthly nurses salary	$4811 ($3608, $6013)	[33-35]
Hourly nurses salary	$27.75 ($20.81,$34.69)	[33-35]
Monthly physicians salary	$16,141 ($12,105, $20,176)	[18,36]
Hourly physician salary	$74.50 ($55.87, $93.12)	[18,36]
Benefits Loading	30% (25%, 35%)	[33,36]
False Positive Rate Elisa Testing	0.005 (0.001, 0.2)	[37,38]
Sensitivity Elisa Testing	0.98 (0.96, 1.0)	[37,38]
False Positive Rate Rapid Testing (Oraquick Advance)	0.002 (0.001, 0.2)	[39]
Sensitivity Rapid Testing (Oraquick Advance – Oral Fluid)	0.993 (0.97, 1.0)	[39]
Elisa cost per test	$4.80 ($3.60, $6.00)	[40]
Western Blot cost per test	$48.50 ($36.38, $60.63)	[40]
Rapid Test cost per test (Oraquick Advance)	$12.00 ($9.00, $15.00)	[39,40]
HIV PCR cost per test	$205.00 ($153.75, $256.25)	[40]
Monthly laboratory staff allocation – Elisa Test	0.17 (0, 0.34)	[18]
Monthly laboratory staff allocation – Western Blot	0.17 (0, 0.34)	[18]
Monthly laboratory staff allocation – HIV PCR	0.17 (0, 0.34)	[18]
Monthly nurse allocation – antenatal care – Elisa test	0 (0, 0.5)	[17,18]
Monthly nurse allocation – labour and delivery – rapid test	0 (0, 0.5)	[17,18,39]
Monthly nurse allocation – Nursery – rapid test	0 (0, 0.5)	[17,18,39]
Pre Test Counseling time – antenatal care (hours)	0.125 (0.094, 0.156)	[17,18,41]
Post Test Counseling time – antenatal care (hours)	0.17 (0.128, 0.213)	[17,18,41]
Pre Test Counseling time – labour or post birth (hours)	0.17 (0.128, 0.213)	[17,18,41]
Post Test Counseling time – labour or post birth (hours)	0.17 (0.128, 0.213)	[17,18,41]
Results Release – HIV- time	0.08 (0.00, 0.10)	[17,18]
Results Release – HIV+ time	0.5 (0.25, 0.75)	[17,18]
# of nurses to be trained – Elisa Test (obstetrics clinic)	32 (24, 40)	[17,34]
# of nurses to be trained – Rapid Test (labour and delivery)	34 (25, 43)	[17,34]
# of nurses to be trained – Rapid Test (nursery)	26 (19, 33)	[17,34]
# of training sessions – Elisa Test	4 (2, 6)	[18]
# of training sessions – Rapid Test	4 (2, 6)	[18]
Length of training – Elisa Test (hours)	4.0 (3.0, 5.0)	[18]
Length of training – Rapid Test (hours)	4.0 (3.0, 5.0)	[18,39]
Training catering costs	$7.50 ($5.00, $10.00)	[18,42]
Investment in audiovisual equipment	$5000 ($3750, $6250)	[42]
Health Promotion activities set up cost	$2,500 ($1875, $3125)	[42]
Annual cost written media items	$3,900 ($2925, $4875))	[42]
Annual cost electronic media items	$25,700 ($19,275, $32,125)	[42]

**Table 2:** Cost Variables (HIV Treatment – Mother) used in model with 'baseline', 'low' and 'high' values, and sources

**Variable**	**Baseline (Low, High)**	**Source**
Antiretroviral medications – pre delivery per month	$600 ($450, $750)	[40]
# of months of medications pre delivery	6 (3, 9)	[2,18]
Antiretroviral Medications – during delivery	$400 ($300, $500)	[40]
Antiretroviral Medications – post delivery per month	$650 ($487.50, $812.50)	[40]
Length Physician consults – pre delivery	0.50 (0.37, 0.63)	[18]
# Physician consults – pre delivery (per month)	1 (0.5,1.5)	[17,18]
Length of pre delivery period (months)	6 (3, 9)	[2,18]
Length Physician consults – post delivery	0.50 (0.37, 0.63)	[18]
# of Physician consults – post delivery (per month)	2.00	[18]
CD4+ cell count – # per month pre delivery	0.33 (0,25 0.5)	[17,18]
CD4+ cell count – # during delivery	1 (0, 1)	[17,18]
CD4+ cell count – # per month post delivery	0.33 (0.25, 0.5)	[18]
Viral load – # per month pre delivery	0.33 (0.25, 0.5)	[17,18]
Viral load – # during delivery	1 (0, 1)	[17,18]
Viral load – # per month post delivery	0.33 (0.25, 0.5)	[18]
FBC # per month pre delivery	0.33 (0.25, 0.5)	[17,18]
FBC # during delivery	1 (0, 1)	[17,18]
FBC # per month post delivery	0.33 (0.25, 0.5)	[18]
Hepatitis A, B, C # per month	0.083 (0, 0.17)	[18]
CD4+ cell count unit cost	$50 ($37.50, $62.50)	[40]
Viral Load unit cost	$205 ($153.75, $256.25)	[40]
Full Blood Count unit cost	$10 ($7.50, $12.50)	[40]
Hepatitis A, B, C unit cost	$50 ($37.50, $62.50)	[40]
Pregnancy termination unit cost	$500 ($200, $700)	[17,43]

**Table 3:** Cost Variables (HIV Treatment – Child) used in model with 'baseline', 'low' and 'high' values, and sources

**Variable**	**Baseline (Low, High)**	**Source**
Prophylactic antiretroviral medications per week	$150 ($112.50, $187.50)	[40]
Duration prophylactic medications (weeks)	6 (4.5,7.5)	[2,18,44]
Duration prophylactic medications (weeks) for false positives	2 (0, 4)	[18]
# of HIV PCR tests – prophylactic period	4 (2, 6)	[18]
# of Elisas performed in prophylactic period	2 (0, 4)	[18]
# of physician consults during prophylactic period	19 (14, 24)	[18,44]
# of physician consultations in prophylactic period for false positives	2 (0, 4)	[18]
Length of physician consult (hours)	0.25 (0.18, 0.31)	[18,44]
CD4+ cell count – # per month HIV+ infant	0.33 (0.25, 0.5)	[18,45]
Viral load – # per month HIV+ infant	0.33 (0.25, 0.5)	[18,45]
Full blood count – # per month HIV+ infant	0.33 (0.25, 0.5)	[18,45]
Confirmation HIV PCR test unit cost	$205 ($153.75, $256.25)	[40]
CD4+ cell count unit cost	$50 ($37.50, $62.50)	[40]
Viral Load unit cost	$205 ($153.75, $256.25)	[40]
Full Blood Count unit cost	$10 ($7.50, $12.50)	[40]
Cost ART for a HIV+ infant per month	$650 ($487.50, $812.50)	[40]
Infant formula cost per month	$81 ($60.75, $101.25)	[44]
# of months formula fed	12 (9, 15)	[44]
Lifetime health care costs of HIV+ infant – normal progressor	$408,375	[30]
Average cost per year – HIV phase	$4063 ($3047, $5078)	[18,30]
Average cost per year – AIDS phase	$13,836 ($10,377, $17,295)	[18,30]
Caregiver costs per year (HIV phase)	$9912 ($7434, $12,390)	[18,31]
Caregiver costs per year (AIDS phase)	$27,604 ($20,703, $34,505)	[18,31]

**Table 4:** Benefit Variables used in model with 'baseline', 'low' and 'high' values, and sources

**Variable**	**Baseline (Low, High)**	**Source**
Percentage of pregnant women unscreened through existing practice	70% (50%, 90%)	[17,18]
Annual population pregnant women	1522 (1142, 1903)	[20]
Percentage pregnant women that accept screening – 14 wks gestation	95% (63%, 100%)	[17,46-48]
Percentage pregnant women that accept screening – onset of labour	85% (67%, 100%)	[17,49,50]
Percentage pregnant women that accept screening – infant at birth	90% (67%, 100%)	[17,50]
Percentage pregnant women that accept ART – 14 wks gestation	75% (50%, 95%)	[18,51]
Percentage pregnant women that accept ART – onset of labour	75% (50%, 100%)	[18]
Percentage of pregnant women that accept ART – infant after birth	75% (50%, 100%)	[18]
Prevalence of undiagnosed HIV in women of childbearing age	4.9% (3.6%, 6.1%)	[18,52]
Additional percentage of screened women who hadn't seroconverted at time of first test or contracted HIV since first test	2% (0%, 6%)	[17,25,50]
% of women who seek antenatal care prior by 14 weeks gestation	63.4% (47.6%, 79.4%)	[17,20]
% of women who seek antenatal care after 14 weeks and present to hospital in sufficient time prior to delivery	25.2% (19.5%, 32.5%)	[17,20]
Termination of pregnancy in diagnosed HIV+ mothers	1% (0%, 5%)	[17,53]
TR without treatment – no breastfeeding	28% (16%, 33%)	[2-4]
TR with treatment from 14 wks gestation- no breastfeeding	2% (1%, 7.3%)	[4,5,54]
TR with treatment from onset of labour – no breastfeeding	10% (5%, 15%)	[4,14,15]
TR with treatment within 24 hours birth – no breastfeeding	13.1% (8.9%, 17.3%)	[15,16]
Life Expectancy HIV- infant	77.74 (58, 97)	[20]
Time to Diagnosis of HIV+ infant without screening (months)	5.2 (3.9. 13.0)	[55,56]
HIV+ infant – % FP	25% (5%, 40%)	[22-24]
HIV + infant – Time in HIV phase NP (screened) (yrs)	8.5 (5.7, 13.5)	[57]
HIV+ infant – Time in HIV phase NP (unscreened) (yrs)	6.2 (4.7, 8.5)	[57]
HIV+ infant – Time in HIV phase FP (screened) (yrs)	0.75 (0.25, 1.25)	[18,57]
HIV+ infant – Time in HIV phase FP (unscreened) (yrs)	0.5 (0.0, 1.0)	[18,57]
HIV+ infant – Time in AIDS phase NP (screened) (yrs)	4.6 (1.7, 10.3)	[18,57]
HIV+ infant – Time in AIDS phase NP (unscreened) (yrs)	3.9 (1.5, 9.7)	[57]
HIV+ infant – Time in AIDS phase FP (screened) (yrs)	0.75 (0.25, 1.25)	[22-24]
HIV+ infant – Time in AIDS phase FP (unscreened) (yrs)	0.5 (0.0, 1.0)	[18,22-24]
Life expectancy HIV+ mother from diagnosis (normal diagnosis path) (years)	12.0 (9.8, 18.2)	[58]
Life expectancy HIV+ mother from diagnosis (early diagnosis due to screening) (years)	12.70 (9.9, 15.0)	[58]
Time to diagnosis of HIV+ mother without screening (months)	20.4 (15.0, 25.5)	[58]

TR = Transmission Rate

FP = Fast Progressor

NP = Normal Progressor

## Results

**Figure 2:**
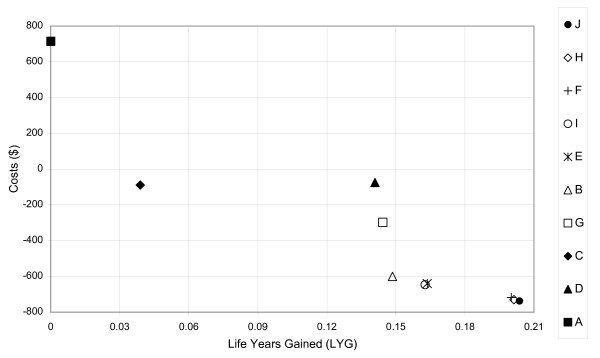
**Cost-Effectiveness Plane – Alternate Strategies for Perinatal HIV Screening – US Virgin Islands**.

With model variables set to baseline values, all strategies generate health benefits and reduce costs when compared to Strategy A which is existing practice. The best alternative is Strategy J, Universal screening by 14 weeks gestation AND screening of the infant at birth. This will save $737.30 and generate 0.2037 life years per pregnant women, per annum. Generalizing to the population of the U.S. Virgin islands of 1522 unscreened pregnant women, the implementation of Strategy J would save $1,122,779 and generate 310 life years. As compared to existing practice, the strategy would save $2,209,564, generate 310 life years, identify an additional 48.14 cases of HIV in mothers and avoid 7.94 cases of HIV in infants. All remaining strategies were dominated by J, that is, they were more costly and less effective. A summary of the results including the cost, effect, incremental cost and incremental effect for each strategy is presented in Table 5. The cost and effect results for each strategy are presented in Figure 2.

**Table 5:** Average and Incremental Cost and Effect Outcomes per Pregnant Woman per Annum

	**Strategy**	**Cost ($)**	**Effect (LYG)**	**Incremental Cost ($)**	**Incremental Effect (LYG)**	**ICER**
J	Universal screening by 14 weeks gestation **and **screening of the infant at birth	-737.70	0.2037			
H	Universal screening by 14 weeks gestation **or **screening at the onset of labour **or **screening of the infant at birth	-730.55	0.2013	7.15	-0.0023	Dominated
F	Universal screening by 14 weeks gestation **or **screening of the infant at birth	-718.82	0.2001	18.88	-0.0036	Dominated
I	Universal screening by 14 weeks gestation **and **screening at the onset of labour	-646.90	0.1627	90.81	-0.0409	Dominated
E	Universal screening by 14 weeks gestation **or **screening at the onset of labour	-639.95	0.1636	97.76	-0.0400	Dominated
B	Universal screening by 14 weeks gestation	-600.54	0.1485	137.16	-0.0552	Dominated
G	Universal screening at the onset of labour **or **screening the infant at birth	-298.17	0.1443	439.54	-0.0594	Dominated
C	Universal screening at the onset of labour	-89.46	0.0389	648.24	-0.1648	Dominated
D	Universal screening of the infant at birth	-75.02	0.1409	662.69	-0.0628	Dominated
A	Sporadic screening	714.05	0.0000	1451.75	-0.2037	Dominated

The finding that Strategy J was optimal is robust to uncertainty in most model parameters. The best decision changed to strategy H when the life expectancy of a HIV positive mother with normal diagnosis (i.e. not diagnosed via the screening strategy) was less than 15.9 years or the rate of HIV transmission from mother to infants without treatment was greater than 19.5%. The results of the microsimulation suggest all strategies were dominated by Strategy J, with Strategy J saving $1079.38 (SD $17,735) and gain 0.2377 (SD 2.32) life years per antenatal member per annum and save $1.6 million and gain 362 life years when the entire unscreened antenatal population is considered.

## Discussion

The results indicate all strategies are cost saving and generate health benefits, even strategies that involve repeat screening. This suggests that a decision not to implement universal screening in a setting where prevalence is high and effective therapies can be delivered is unethical. Excess costs will be incurred and life years lost at the same time.

The strategy with the greatest saving and the greatest LYG is Strategy J, universal screening by 14 weeks gestation AND screening of the infant at birth. This will inform the mother of her HIV status if she was not screened by 14 weeks and update the mother if she was screened earlier as some women may have contracted the virus during their pregnancy and some may have not seroconverted at the time of their initial screening. Strategy J was robust to most changes within the model, including a 5% discount rate for future costs and benefits, but not all. When 'life expectancy of a HIV+ mother with normal diagnosis' took a value of 15.9 years or greater, Strategy J loses its dominance to Strategy H.

The conclusion also changes when alternate values for the rate of transmission with no treatment are applied. For lower values, Strategy H is the optimal strategy. For higher values, Strategy J is the optimal strategy. Previous studies have also found that their results have been sensitive to the efficacy of treatment but the likelihood that the transmission rate is as low as 19.5% is minimal [12,25]. While transmission rates have been reported as low as 13%, the majority of the studies report transmission rates greater than 25% [26].

In addition to providing clear policy advice, this study is novel due to the comprehensive range of screening options evaluated. The authors of most studies only evaluate screening women once, at one point in time [8,12,27-29]. The authors of only three studies have compared screening women at more than one point in time [22-24] and none have compared the 'AND' strategies (i.e. strategies I and J) included in this evaluation. The conclusion that repeat screening is cost-effective is therefore novel. The model structure also allows the impact of different rates of disease progression in the infant [22-24], the additional benefits to the mother and infant from early diagnosis, the cost of caring for a child with HIV and AIDS [30,31], the medical costs of a child with HIV and AIDS and the impact of rapid testing [18].

There are several limitations to this research that warrant further investigation. There is little research into the acceptance of a blood specimen as opposed to a saliva specimen for rapid testing and this may affect compliance to testing. Little is known about whether the acceptance of screening may differ between different races, ethnicities and socioeconomic groups. There is also little evidence of the acceptance of HIV treatment recommendations by a mother including the acceptance of treatment for her HIV+ infant and avoiding breastfeeding her HIV+ or HIV- infant. There is the potential that further testing of the mother and/or infant may be required to assess compliance which is not addressed in this study. In addition, the literature indicates that the earlier a woman is diagnosed and ART commenced, the greater the potential to avert virus transmission to the infant. In this study, if a woman does not attend prenatal care by 14 weeks gestation, the next time she would be offered screening is at the onset of labor. There would be a greater potential to avert virus transmission for those who do not attend prenatal care by 14 weeks if they were offered screening earlier than at the onset of labor. These potential additional benefits are not addressed in this study. There is limited data for any population on the incidence of women who seroconvert after the time of their initial test or the incidence of women who contract HIV after their initial screen. This information would be of importance in reinforcing the benefits of offering screening at during more than just the prenatal visit before 14 weeks gestation. Our understanding of the life expectancy of a HIV positive infant, the lifetime health care costs of a HIV positive infant and the treatment regimes for children are constantly changing. Such changes often mean that the previously published literature does not truly reflect the effectiveness of current practice. While we believe that our high and low values for these variables are reasonable and our conclusions are robust to these changes, the continuing improvements in clinical practice should be monitored to ensure that the values for these variables are as reflective as possible of the true cost and effectiveness of current treatment. While the results of the microsimulation demonstrate possible variation in population characteristics, the value of a variable for each simulation is chosen randomly on all levels. Had probability distributions been fitted to model parameters then second order (parameter) uncertainty could have been characterized and cost-effectiveness acceptability curves fitted. This framework also allows the value of reducing uncertainty in model parameters to be estimated, thus providing a valuation of further data collection in the future.

This study highlights that the decision of the U.S. Virgin Islands to adopt standard U.S. policy for perinatal HIV screening is inefficient. Women need to be offered screening more than once and at different points in time. A change to this policy, we suggest, would represent an excellent public health investment, saving nearly 310 life years with an ultimate costs saving of over $1.1 million when the entire antenatal population is considered. The study has not only addressed the unique characteristics of the U.S. Virgin Islands but has addressed the cost-effectiveness of antenatal HIV screening for a lower high income country, a country with relatively high HIV prevalence yet relatively substantial resources available to be dedicated to health care. To date, other studies have addressed either high income countries [8,9,25,29,32], or low or lower middle income countries [10,11] but their recommendations are generally not applicable to upper middle income or lower high income countries. These countries may not have the resources to implement strategies recommended for upper high income countries as the range of countries classified as high income is very broad, yet can do more than those recommendations for low income or lower middle income country. Puerto Rico is one country that these results might be generalized to. It is a U.S. Territory that adopts much of its healthcare policy from the mainland U.S. Like the U.S. Virgin Islands, it is a lower upper middle income country with resources available for healthcare. The two countries have similar rates of antenatal care, HIV prevalence greater than that of the mainland U.S. and cases of vertically transmitted HIV are still being diagnosed. Furthermore, both countries are in similar geographical regions with similar population characteristics with Puerto Rico having a higher portion of its population of Hispanic ethnicity. There are several other countries that these results may be generalisable to. While none are as similar in characteristics as Puerto Rico, a country with a similar gross domestic product, mainly upper middle and lower high income countries might benefit from this research owing to the fact that these countries may not have the resources available to implement screening strategies recommended for upper high income countries, yet can do more than the recommended strategies for low income countries. Countries that may fall into this category include other U.S. Territories (Guam and American Samoa), other Caribbean countries (The Bahamas, Aruba, Barbados, St Lucia, St Kitts and Nevis) and even countries within the Americas and Europe (Mexico, Hungary and the Czech Republic). In order to generalize, consideration would need to be given to HIV prevalence in women of child bearing age, the rates of prenatal care in the country, acceptance of screening, population and cultural characteristics and the pricing and availability of treatment. Furthermore, the incremental analysis of this study only accounts for the new inputs that are required and does not consider the total costs by including the costs of the existing program. To ensure generalisability between settings, existing program costs, including infrastructure, of the settings would need to be accounted for.

## Conclusion

This study demonstrates the inefficiency of adopting standard perinatal HIV screening policies without addressing a regions population characteristics, HIV prevalence, and the resources available for healthcare. The conclusions provide an opportunity to develop clear, unambiguous and practical policy that will drive costs downward and deliver increased health benefits. This represents an improvement in economic efficiency and a positive step toward treating a serious and prevalent disease. Additionally, this study highlights the benefits of offering screening at different opportunities and of repeat screening. The findings of this study are not only useful to the U.S. Virgin Islands, but raise the question of the generalisability of these results to countries that share similar economic, epidemiological and social characteristics.

## Abbreviations

ART: Antiretroviral Therapy; EIA: Enzyme Linked Immunoassay; HIV: Human Immunodeficiency Virus; ICER: Incremental Cost Effectiveness Ratio; LYG: Life Year Gained; PCR: Polymerase Chain Reaction; TR: Transmission Rate; US: United States of America; U.S. Virgin Islands: United States Virgin Islands.

## Competing interests

The authors declare that they have no competing interests.

## Authors' contributions

BU developed the economic model, reviewed and collected data, performed the analysis and drafted the manuscript. NG contributed to the development of the economic model, performing of the analysis and drafting the manuscript. CU contributed to the development of the economic model and the review and collection of data. All authors read and approved the final manuscript.

## Pre-publication history

The pre-publication history for this paper can be accessed here:


